# Preoperative administration of etoricoxib in patients undergoing hip replacement causes inhibition of inflammatory mediators and pain relief

**DOI:** 10.1002/j.1532-2149.2011.00062.x

**Published:** 2011-12-19

**Authors:** B Renner, G Walter, J Strauss, MF Fromm, J Zacher, K Brune

**Affiliations:** 1 Institute of Experimental and Clinical Pharmacology and Toxicology, University of Erlangen-NürnbergErlangen, Germany; 2 Department of Orthopedics, HELIOS Klinikum Berlin-BuchBerlin, Germany; 3 Department of Anesthesiology, HELIOS Klinikum Berlin-BuchBerlin, Germany

## Abstract

**Objective:**

Administering cyclooxygenase-2 inhibitors preoperatively appears attractive since these drugs reduce post-operative pain, but do not increase the risk of post-operative bleeds, asthmatic attacks and stress-related gastrointestinal ulcers. In a former investigation, we could show that post-operative administration of etoricoxib reduces prostaglandin production in wound fluid, but the onset of action is variable due to delayed post-operative absorption.

**Methods:**

In this study, we investigated the preoperative administration of etoricoxib in patients undergoing hip replacement. They received 120 mg etoricoxib or placebo 2 h before surgery and 1 day after in a double-blinded, randomized, parallel group design.

**Results:**

A total of 11 patients were randomized (placebo *n* = 5; verum *n* = 6). We found high and constant levels of the drug in blood, central nervous system and wound fluid already at the end of surgery (t_max_ < 2 h). This was accompanied by inhibition of prostaglandin production in the wound tissue (treatment *p* < 0.05), suppression of interleukin 6 increase in plasma (treatment *p* < 0.01), and – despite existing standard pain relief procedures – higher satisfaction with analgesics (time vs. treatment *p* < 0.05) and less demand for opioids (treatment *p* < 0.01) and intrathecal bupivacaine (treatment *p* = 0.05) administration.

**Conclusion:**

Administration of etoricoxib 2 h before surgery allows for an effective drug concentration in critical tissues, a reduction of the production of pro-inflammatory mediators and for better pain relief.

## 1. Introduction

Major surgery requires instantaneous post-operative pain relief. Opiates and opioids, given during and after surgery, reduce post-operative pain. Epidural co-administration of local anaesthetics is regarded useful, but complete satisfaction is often not achieved (Brattwall et al., 2010). In addition, these measures may cause delayed mobilization of the patients and retarded normalization of bowel movements. Moreover, respiration and – going along with it – blood oxygenation may be inadequate (Perttunen et al., 1992).

Several reports indicate that the additional administration of cyclooxygenase (COX) inhibitors may reduce post-operative pain (Perttunen et al., 1992; Brattwall et al., 2010). Traditional non-steroidal anti-inflammatory drugs (NSAIDs; non-selective COX inhibitors) are often contraindicated due to their inhibition of blood coagulation (Marret et al., 2003; Li et al., 2009), risk of gastrointestinal (GI) ulcerations and attacks of aspirin inducible asthma. Among the available cyclooxygenase-2 (COX-2) selective inhibitors, celecoxib appears less adequate for preoperative administration due to its slow and incomplete absorption (Brune et al., 2010). Parecoxib may be given, but only i.v. post-operatively. Etoricoxib is used for this purpose frequently in several countries (Clarke et al., 2009), but it lacks the indication for treatment of ‘postoperative pain’. It may be used, however, as inhibitor of heterotopic ossification (Sodemann et al., 1990).

Previously, we aimed at defining the pharmacokinetics of etoricoxib in patients having undergone hip replacement (Renner et al., 2010). We demonstrated that the pharmacokinetic and pharmacodynamic (PK/PD) profile of etoricoxib given 1 day after surgery is comparable to that observed in healthy volunteers. However, onset of absorption was variable, and the contribution to pain relief on top of the standard post-operative pain therapy using opioids and/or local anaesthetics could not be assessed as the standard pain therapy did not leave much space for further improvement 2 days after surgery when pain was less prominent. In this study, we aimed at evaluating the merits of preoperative administration of 120 mg etoricoxib 2 h before and 1 day after surgery in a placebo-controlled, double-blinded and parallel group design.

## 2. Methods

After approval from the German authorities and the Institutional Ethics Review Board, 11 male and female patients (aged 59–77 years) with osteoarthritis undergoing elective primary single hip arthroplasty were consented. All patients were recruited at the Department of Orthopedics, HELIOS Klinikum Berlin-Buch, Germany. The clinical trial is registered at EudraCT (#2005-003854-80) and at ClinicalTrails.gov (#NCT00746720). The study was conducted according to the Declaration of Helsinki on biomedical research involving human subjects (Somerset West amendment). All patients gave their informed consent prior to their inclusion in the study. One patient was excluded from the study on day 2 because the intrathecal (IT) catheter was removed by mistake. In a further patient, cerebrospinal fluid (CSF) samples could only be recorded on days 1 and 2 due to technical reason (catheter occlusion) and concomitant aspirin intake (100 mg orally). In one patient, there was an adverse event (nausea) on day 4 which could be treated successfully with 20 mg metoclopramide (p.o.). A causal relationship to the study drug 2 days after the last administration was considered unlikely. Since only 11 out of 40 planned patients could be recruited due to administrative and personal changes, the investigators decided to terminate the study in advance.

### 2.1 Patients and study design

Exclusion criteria were: renal insufficiency (serum creatinine > 1.5 mg/dL), recent major trauma or systemic infection (within 3 months), history of usage of corticosteroid medication or chronic opioids (within 3 months), conditions likely to affect prostaglandin levels and conditions contraindicating spinal anaesthesia. In addition, patients were excluded if they had the following characteristics: hypersensitivity to any component of the study medication; uncontrolled hypertension during rest at two repeated measurements; congestive heart failure (New York Heart Association II-IV); cerebrovascular disease; established ischaemic heart disease (including patients who had recently undergone coronary artery bypass graft surgery or angioplasty); elevated liver function enzymes (threefold above normal range); patients who had developed signs of asthma, acute rhinitis, nasal polyps, angioneurotic oedema or urticaria following the administration of acetylsalicylic acid or other NSAIDs; pregnancy and lactation; patients with active peptic ulcerations or active GI bleeding; and inflammatory bowel disease.

Preoperative demographic data were collected and all preoperative medications including dose, route and duration were recorded. Pain scores were assessed using a numerical rating scale (11-point category scale: 0 = no pain, 10 = worst possible pain) (Downie et al., 1978). The global (overall) pain and the specific pain from the replaced hip at rest and passive movement were obtained.

The study allowed for only a limited number of (fluid) samples over a period of 48 h due to safety reasons (overall sampling volume for CSF and plasma). All patients received the spinal anaesthetic bupivacaine 0.5% (7.5–10 mg) via an IT catheter placed at the lumbar 3–4 or 4–5 interspace. They were maintained at normothermia in the operating room by supporting means, including warmed intravenous fluids. Post-operatively, 0.01% bupivacaine was infused intrathecally at a continuous rate superimposed by patient controlled IT boluses of bupivacaine. Initial basal infusion rate was 4–6 mL/h with patient-controlled boluses of up to 1 mL q 10 min and a 4-h lockout of 40 mL. The patients were allowed to titrate IT requirements for analgesia using a previously applied analgesic efficacy protocol (Buvanendran et al., 2003). Piritramide was given i.v. at a dose up to 5 mg (subcutaneously up to 15 mg) for breakthrough pain. Total drug consumption was recorded. If patients presented with increased body temperature (> 39 °C), oral acetaminophen 1.0 g was administered after contact with one of the investigators. A standardized surgical technique of non-cemented hip arthroplasty was used for all patients.

**Figure 1:**
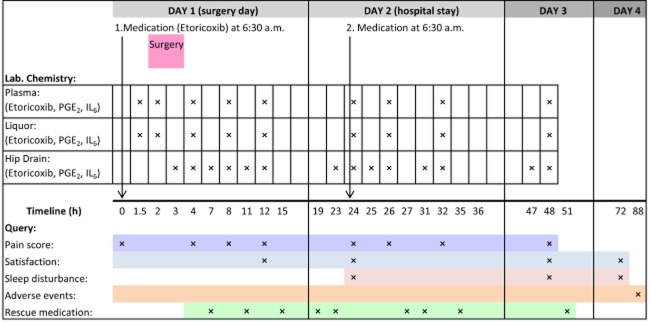
Study time schedule with four data recording days. Medication time on surgery day was set as time 0 h. IL6, interleukin 6; PGE_2_*,* prostaglandin E_2_.

On the day of surgery and the day after, six patients received oral etoricoxib 120 mg at 6:30 a.m. and five patients received placebo. The study was blinded to patients and the investigators, but not to the analytical department. Blood (3 mL) and CSF samples (0.5 mL) were collected before and at the following time points after drug administration: 1.5, 2, 4, 8, 12, 24, 26, 32 and 48 h (nine time points, see Figure 1). The initial 1 mL of each CSF sample was discarded to account for dead space in the catheter and to avoid possible dilution artefacts regarding the intrathecally applied bupivacaine. The post-operative analgesic IT solution was administered to provide continuous analgesia for the patients between sampling time points.

Tissue exudates (hip drain) were collected at 4, 8, 12, 24, 26, 32 and 48 h after drug administration. One hour before the scheduled collection time, the Redon drain system (B. Braun AG, Melsungen, Germany) was emptied and the exudate was collected over the next 60 min (additional sampling times at 3, 7, 11, 23, 25, 31 and 47 h post medication, see Figure 1). Drain fluids were centrifuged and the supernatant was frozen at −30 °C and sent for analysis.

### 2.2 Pharmacokinetic analysis

Following the oral administration of etoricoxib, pharmacokinetic parameters were determined in plasma, tissue exudate and CSF in each subject using a liquid chromatography-tandem mass spectrometry (LC-MS/MS). The analytical method was adapted from published work on plasma using phenazone as an internal standard (Bräutigam et al., 2003). The detection was performed on an API 4000 (Applied Biosystems, Langen, Germany) and determination of the limits of quantification (LOQ) for etoricoxib plasma, CSF and tissue exudates was carried out according to food and drug administration (FDA) guidelines (US Department of Health and Human Services, 2001). The LOQ for etoricoxib was 0.2 ng/mL in all three matrixes. The analytical method was validated over the concentration range 0.2–200 ng/mL (Renner et al., 2010). The area under the curve (AUC) [ng·h/mL] from 0 to 24 h was calculated for both CSF and plasma data using the linear trapezoidal method (Phoenix WinNonlin 6.1; Pharsight Corp., MO, USA). The fraction of plasma etoricoxib in the CSF was calculated from the ratio of AUC_CSF_ to AUC_plasma_ at each sampling time.

Prostaglandin E_2_ (PGE_2_) and interleukin 6 (IL6) analyses from all three matrixes (except PGE_2_ analysis from CSF) were performed using enzyme-linked immunosorbent assay kits (Cayman Chemical, MI, USA). The LOQ for PGE_2_ was 15 pg/mL and that for IL6 was 7.8 pg/mL. CSF samples were analysed for PGE_2_ using LC-MS/MS methods, which were developed in our prior study using a stable isotope of prostaglandin D_2_-d_4_. The solid phase extraction method and the LC-MS/MS conditions were adjusted to our API 4000 system using previous publications (Nithipatikom et al., 2003; Schmidt et al., 2005). The lower LOQ was 25 pg/mL and the upper LOQ was 200 pg/mL according to FDA guidelines (US Department of Health and Human Services, 2001). The cytokine concentrations at the central (CSF) and peripheral (plasma and tissue exudate) sites over time were compared between the study groups.

### 2.3 Secondary parameters

Pain scores at rest and passive movement of the operated hip were assessed at 4, 8, 12, 24, 26, 32 and 48 h time points after study drug administration (see Figure 1). The total IT medication consumption, the number of patient-activated requests, delivered boluses and time of requests were documented. Tympanic temperature was monitored at the preoperative visit and at each time point of blood and CSF sampling.

The haematological status comprised a complete cell count (erythrocytes, leukocytes and platelets). It was obtained pre- and post-operatively. Blood products transfused during the perioperative phase were recorded. In addition, coagulation parameters were measured and recorded (pre- and post-operatively). Patients rated their sleep disturbance due to pain at 24, 48 and 72 h after surgery (0 = no sleep disturbance to 10 = maximal sleep disturbance; see Figure 1).

### 2.4 Statistical analysis

Demographic and other univariate data were analysed using *t*-tests, χ^2^ tests, and Fisher's exact tests, or the Mann–Whitney *U*-test, dependent on the scale and distributional characteristics of the variables.

Outcomes with measures at multiple time points were calculated as partial AUCs in order to generate comparable time periods and to account for the small number of patients. Partial AUCs were calculated from time point of first measure to 12 h, from 12 to 24 h, from 24 to 36 h and from 36 to 48 h (or time point of last observation) using the linear trapezoidal method. A linear mixed model for repeated measures was used to fit and analyse the data. ‘Time’ (AUC 1–4), ‘treatment’ (placebo vs. etoricoxib) and ‘treatment by time’ interaction were the terms in the model. In an explorative analysis, the between-subject factor ‘gender’ was introduced to account for evident sex differences in the data. *T*-tests with Bonferroni-adjusted *p*-values were used for pair-wise comparisons between treatment groups. Therefore, not significant values (*p* ≥ 0.05) are also given in figures and tables. All statistical analyses were performed using the IBM SPSS Statistics program package (version 19.0.0.1 for Windows™; SPSS Inc., Chicago, IL, USA). Sample size calculations were performed using the software package GPOWER (version 2.0; Faul, F. & Erdfelder, E., Bonn University, Bonn, Germany).

## 3. Results

The results of this cohort study are given in the figures and tables. The small size of our cohort did not allow for perfect randomization. The patients in the verum group were slightly older, lighter and consisted of women only, while the placebo group included both sexes (Table 1). Otherwise, the groups were homogenous. The time schedule of the investigation is depicted in Figure 1. We took care to have all parameters recorded for at least 3 days.

**Table 1:** Demographic characteristics of the patients

	Placebo (*n* = 5) Mean (SD)	Etoricoxib (*n* = 6) Mean (SD)	*p*-value
Age (year)	68.2 (6.6)	69.3 (6.3)	0.778
Weight (kg)	96.6 (21.4)	77.2 (6.1)	0.112
Height (cm)	174 (7)	163 (6)	**0.023**
Sex (counts)			
Female/male	2/3	6/0	0.061^a^
Race (counts)			
Caucasian	5 (100%)	6 (100%)	ND
Duration of surgery (min)	40 (11)	46 (22)	0.603
Intra-OP BL (mL)	450 (187)	383 (98)	0.500

Intra-OP BL, blood loss during surgery; ND, not done (statistic cannot be computed); SD, standard deviation.

Values are mean (standard deviation) unless otherwise indicated.

a Fisher's exact test due to low counts *p*-value may be biased.

**Figure 2:**
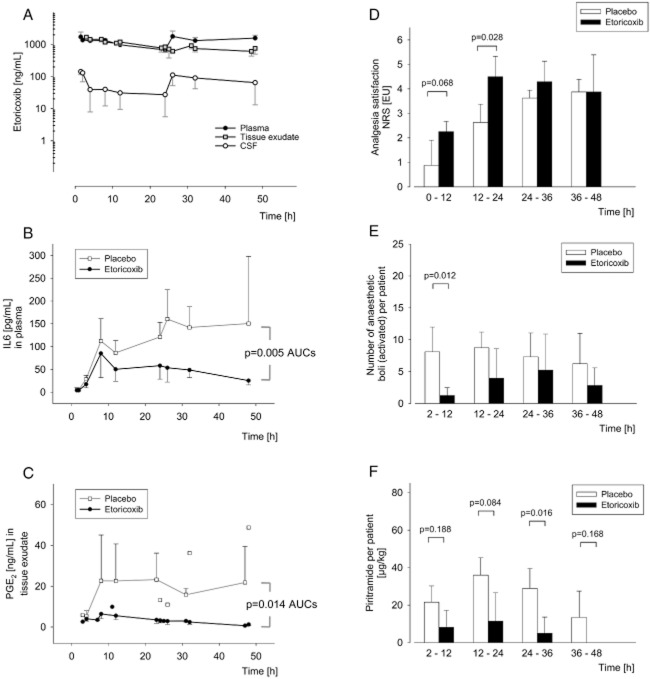
(A) Etoricoxib concentration time courses in plasma, cerebrospinal fluid (CSF) and tissue exudates (hip drain) after administration of 120 mg at time 0 and 24 h [means ± standard deviation (SD); *n* = 6; see Table 2]. (B) Interleukin 6 (IL6) plasma concentrations in the placebo and etoricoxib group (significant group differences, see Table 3; means ± SD; *n* = 10–11). (C) Prostaglandin E_2_ (PGE_2_) concentrations in tissue exudates comparing placebo and verum group (means ± SD; *n* = 10; data with *n* = 1 are shown as single dots and are not included in the line graph; post hoc comparisons, see Table 3). (D) Patient estimates for satisfaction with post-surgery analgesia recorded on a numeric rating scale (NRS) with 0 = no efficacy to 5 = excellent efficacy (means ± SD; *n* = 10). (E) Number of patients’ requests for intrathecal (IT) local anaesthetic (bupivacaine) boli per patient (means ± SD; *n* = 9–10). (F) Opioid analgesic (piritramide) administration per patient in the etoricoxib and placebo group (means ± SD; *n* = 10). (D–F) Since the IT catheter for applying bupivacaine was absent in one patient in the placebo group, this patient was excluded from pain-related statistical analysis and from graph data (significant group differences are presented in graphs with Bonferroni adjusted *p*-values). AUCs, areas under the curve.

We observed high concentrations of etoricoxib in plasma and CSF immediately after surgical intervention in all patients treated (Figure 2A). The concentrations of etoricoxib decreased slowly after drug administration, i.e., during the two following days with an increase after the second dose 24 h after the initial one (Figure 2 and Table 2). In line with this observation, we found a lasting post-operative increase in IL6 in the plasma of the placebo group. In the etoricoxib group, IL6 values returned to preoperative levels within 20 h after surgery (Figure 2 and Table 3). Statistical analysis revealed a significant ‘overall treatment’ (*p* = 0.005) and ‘time’ (*p* = 0.033) effect and a possible interaction (time vs. treatment: *p* = 0.073). Similarly, PGE_2_ concentration in wound fluid remained low throughout (Figure 2C and Table 3) in contrast to the placebo group where a fast increase during the first hours after surgery was followed by a high and increasing plateau. Correspondingly, we observed significant main effects for ‘treatment’ (*p* = 0.014) and ‘time’ (*p* = 0.016) together with a significant interaction (time vs. treatment: *p* = 0.012). The effects of etoricoxib administration on tissue and CSF IL6 concentrations were less clear. A ‘leave one out analysis’ (not quantifiable IL6 samples from CSF up to 24 h in this subject) revealed a significant ‘time’ (*p* = 0.003) effect and ‘time versus treatment’ interaction (*p* = 0.030) for IL6 in the CNS. The overall differences between verum and placebo did not achieve statistical significance (Table 3). Tissue IL6 concentrations changed significantly over time (*p* < 0.001), but the ‘treatment’ effect failed to reach statistical significance (effect treatment: *p* = 0.073; interaction: *p* = 0.084; descriptive data: see in Table 3). A subsequent sample size calculation indicated that at least 24 subjects would have had to be included in order to detect treatment effects at a power level above 80% (12 in each treatment group).

**Table 2:** Pharmacokinetic parameters of etoricoxib comparing CSF and plasma data

	Plasma	CSF
AUC _0–24 h_ [h·ng/mL]^a^	26,008 (4161)	1106 (439)
AUC _0–48 h_ [h·ng/mL]^a^	60,979 (11,401)	2904 (1303)^b^
t_max_ [h] _(0–24 h)_^c^	1.5 (1.5–8)	1.5 (1.5–2)
c_max_ [ng/mL] _(0–24 h)_^a^	1884 (773)	160 (42)
CSF/plasma ratio AUC _0–24 h_ [%]^d^	4.1 (1.7–5.8)	
CSF/plasma ratio AUC _0–48 h_ [%]^d^	4.7 (2.8–8.1)^b^	
t_max_ [h] for CSF/plasma conc. ratio^c^	1.75 (1.5–32)	
CSF/plasma conc. ratio [%] at 1.5 h^a^	9.7 (4.5)	
Maximal CSF/plasma conc. ratio [%]1	12.2 (3.7)	

AUC, area under the curve; conc., concentration; CSF, cerebrospinal fluid; SD, standard deviation.

a Mean (± SD).

b Median (range).

c Individual CSF/plasma ratios were calculated using AUC_0–24 h_ or AUC_0–48 h_; mean (range) data are given in percentage values.

d Subgroup analysis without data from one patient due to incomplete CSF samples (*n* = 5).

e Maximal CSF/plasma ratios were calculated using individual maximal concentration ratio (mean ± SD); data are given in percentage values.

**Table 3:** Effects of etoricoxib given pre- and post-operatively on pain-related mediators (post hoc pair-wise comparisons with Bonferroni adjustments)

	Placebo				Etoricoxib				
			
	Mean	SD	*n*	SE	Mean	SD	*n*	SE	*p*-value
IL6 plasma									
AUC (1.5–12 h) [h·pg/mL]	714	287	5	128	435	307	6	125	0.624
AUC (12–24 h) [h·pg/mL]	1244	371	5	166	596	339	6	139	**0.056**
AUC (24–36 h) [h·pg/mL]	1619	343	4	171	598	288	6	118	**0.004**
AUC (36–48 h) [h·pg/mL]	1768	1231	4	615	409	154	6	63	0.100
PGE_2_ tissue exudate									
AUC (4–12 h) [h·ng/mL]	125	134	4	67	40	15	6	6	0.616
AUC (12–24 h) [h·ng/mL]	227	182	4	91	48	21	6	9	0.160
AUC (24–36 h) [h·ng/mL]	239	139	4	69	29	13	6	5	**0.020**
AUC (36–47 h) [h·ng/mL]	278	184	4	92	16	9	6	4	**0.028**
IL6 cerebrospinal fluid (w/o R111)									
AUC (1.5–12 h) [h·pg/mL]	835	807	4	403	340	241	6	98	0.744
AUC (12–24 h) [h·pg/mL]	2050	1633	4	816	660	614	6	251	0.224
AUC (24–36 h) [h·pg/mL]	644	418	3	242	575	456	5	204	1.000
AUC (36–48 h) [h·pg/mL]	362	245	3	141	436	373	5	167	1.000
IL6 tissue exudate									
AUC (4–12 h) [h·ng/mL]	523	275	4	137	479	327	6	133	ND
AUC (12–24 h) [h·ng/mL]	2107	975	4	488	1606	1221	6	499	ND
AUC (24–36 h) [h·ng/mL]	2566	1166	4	583	1200	838	6	342	ND
AUC (36–47 h) [h·ng/mL]	2393	1272	4	636	861	503	6	205	ND

ND, post hoc comparison was not done because neither the overall treatment effect nor the interaction (time vs. treatment) was statistically significant. AUC, area under the curve; IL6, interleukin 6; PGE_2_, prostaglandin E_2_; SD, standard deviation; SE, standard error; w/o, without. Bold p-values indicate therapeutic advantages (significant or values with tendency).

**Figure 3:**
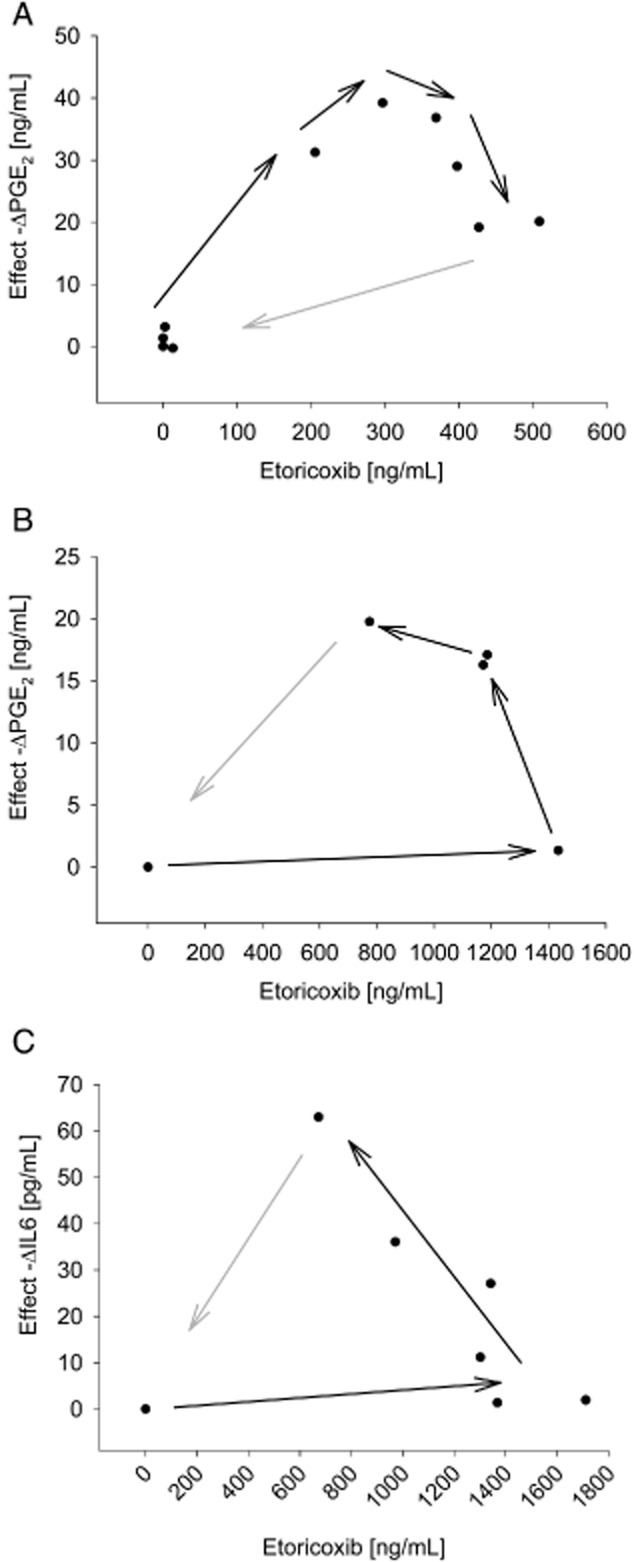
Pharmacokinetic and pharmacodynamic (PK/PD) relationship and pre- versus post-operative dosing effects: Local drug concentrations versus corresponding inhibitory effects on inflammatory mediators during the first 24 h after administration of 120 mg etoricoxib are presented. The effect size was derived from mean group differences with positive values indicating inhibitory effects on inflammatory mediators. Arrows specify the time course of the relationship. (A) Tissue exudate data for prostaglandin E_2_ (PGE_2_) were derived from our previous study (Renner et al., 2010) and showed a direct PK/PD relationship during drug accumulation. At later time points, the effect decreased in a clockwise hysteresis. Note: The drug was administered 20 h post-operatively allowing PGE_2_ to increase after surgery in both groups. (B–C) Tissue exudate data for PGE_2_ (B) and plasma interleukin 6 (IL6) (C) from the present study with a counterclockwise relationship indicating for a delayed drug response. Dosing time before surgery resulted in a delay of inhibitory effects (counterclockwise hysteresis).

The explorative inclusion of the factor ‘gender’ revealed no obvious effect on both inflammatory mediators tested (gender: range for *p* = 0.155–0.865). In Figure 3, differences in inflammatory mediators between both treatment groups are plotted versus the corresponding etoricoxib concentrations in order to characterize a PK/PD relationship for the significant parameters (Figure 3B and C). Related tissue PK/PD data from our previous study are depicted in Figure 3A.

In line with these findings, we observed significantly higher post-operative analgesic satisfaction in the patients receiving etoricoxib as compared with those in the placebo group during the first 24 h (main effect treatment: *p* = 0.083; time: *p* < 0.001; interaction: *p* = 0.036; Figure 2D). At days 3 and 4, the differences between the two groups disappeared. Congruent with this observation, we found that on request local anaesthetic administration was higher in the placebo group but only during the first 12 h post-operatively. However, the overall treatment effect *p*-value was slightly above limit (main effect treatment: *p* = 0.052; time: *p* = 0.043; Figure 2E). The explorative inclusion of the factor ‘gender’ improved to some extent the ‘treatment’ effect (treatment: *p* = 0.027; gender: *p* = 0.164). Similarly, the demand for piritramide was significantly higher in the placebo group (main effect treatment: *p* = 0.003; time: *p* < 0.001; interaction: *p* = 0.067). This difference is visible from 2 to 48 h and reached statistical significance at the second day (24–36 h post study medication). It was not statistical significant at day 3 (Figure 2F). As before, the explorative analysis for gender effects on these pain-related data did not hint at any evident effect (gender: range for *p* = 0.164–0.656).

The concentrations of PGE_2_ in plasma and CSF were near or below the LOQ in both groups. The differences did not reach statistical significance. Similarly, the effects of etoricoxib administration on other parameters, including pain at rest, pain at movement, etc., showed a trend in favour of etoricoxib administration, but did not reach significance (compare Table 4).

**Table 4:** Patient responses to pre- and post-operative administration of etoricoxib (non-significant parameters)

		Placebo				Etoricoxib			
			
	Time	Mean	SD	*n*	SE	Mean	SD	*n*	SE
Global pain									
0 = no pain	Day 1	2.0	1.3	5	0.6	**1.5**^a^	1.3	6	0.5
10 = worst pain	Day 2	2.8	0.8	4	0.4	**1.4**	1.4	6	0.6
Global pain at rest									
0 = no pain	Day 1	2.7	1.1	5	0.5	**1.6**	0.9	6	0.4
10 = worst pain	Day 2	1.2	0.6	4	0.3	**1.0**	0.9	6	0.4
Pain at passive movement									
0 = no pain	Day 1	4.6	1.3	5	0.6	**3.8**	1.1	6	0.5
10 = worst pain	Day 2	4.8	2.4	4	1.2	**3.6**	2.3	6	1.0
Overall satisfaction									
1 = poor	Day 1	2.2	1.0	5	0.4	**2.8**	0.4	6	0.2
2 = fair	Day 2	**2.8**	0.4	4	0.2	2.3	0.7	6	0.3
3 = good	Day 3	**2.8**	0.4	4	0.2	2.7	0.7	6	0.3
	Day 4	2.8	0.4	4	0.2	**3.0**	0.0	5	0.0
Sleep disturbance									
0 = no	Day 1	**3.2**	1.9	5	0.9	5.3	3.9	6	1.6
10 = great	Day 2	4.3	2.9	4	1.4	**2.8**	2.0	6	0.8
	Day 3	3.8	3.3	4	1.6	**3.2**	1.3	5	0.6
IT bupivacaine									
Total volume [mL]	Days 1–2	291.9	32.9	4	16.4	**226.0**	66.4	6	27.1
Delivered boli per patient	2–6 h	1.8	1.7	4	0.9	**0.4**	0.5	6	0.2
	6–24 h	3.3	0.7	4	0.4	**2.3**	2.5	6	1.0
	24–48 h	2.8	2.7	4	1.4	**1.1**	1.1	5	0.5

IT, intrathecal; SD, standard deviation; SE, standard error.

a Bold values indicate therapeutic advantages.

## 4. Discussion

The use of non-opioid analgesics (COX inhibitors) in the post-operative setting has a long tradition (Moiniche et al., 2002). Their preoperative administration was always limited due to the risk of post-operative bleeds (Moiniche et al., 2003). COX-2 selective inhibitors are devoid of this risk. They are therefore attractive analgesics in the perioperative setting (Clarke et al., 2009). Presently, it is unclear if preoperative administration should be preferred against (early) post-operative administration (Moiniche et al., 2002). Moreover, as the two COX-2 inhibitors available for oral use (namely celecoxib and etoricoxib) differ in their PK/PD profile (Brune et al., 2010), both should be investigated individually on the basis of their pharmacokinetic characteristics. Etoricoxib shows fast absorption, high bioavailability and slow elimination (Agrawal et al., 2003). Therefore, preoperative administration should guarantee instantaneous and lasting post-operative effects. Consequently, our study aimed at defining the PK/PD relations between etoricoxib absorption and distribution and the inhibition of the production of mediators of inflammation, pain and overall (analgesic) satisfaction. Our pharmacokinetic results for etoricoxib were comparable to previous findings (Renner et al., 2010). However, in the present study we found that preoperative administration of the drug guaranteed stabile concentrations in critical tissues, i.e., plasma, CSF and wound fluid, immediately after surgery. This led to complete blockade of PGE_2_ production in the surgical wound and a reduced release of IL6 into plasma. This in turn corresponded to higher satisfaction of the patients and reduced demands for post-operative opioids and IT local anaesthetics.

An additional benefit for patients in the etoricoxib group is likely. There is a trend for other parameters (compare Table 4), as, e.g., pain at rest or pain at movement, in favour of etoricoxib administration. It did however not reach statistical significance which is not surprising in view of the small sample size. Unwanted drug effects were not seen in either treatment group. Only one adverse event on study day 4 (nausea) was recorded. A causal relation to the study drug was rated unlikely (2 days after placebo administration).

Patient recruitment had to be closed in advance due to personal and administrative changes. Therefore, the data management and statistical analysis had to be adjusted to the small number of patients. Therefore, we aggregated measures at multiple time points into time frames of 12 h (see Methods). Also, our study design was not intended to detect gender differences nor was the sample size adequate for such a calculation. Nevertheless, we performed an exploratory confounder analysis which did not reveal any obvious gender effect with respect to inflammatory and pain-related data.

Etoricoxib has been suggested as a co-analgesic in perioperative use in connection with tooth extraction (Malmstrom et al., 2004), extirpation of the uterus (Chau-in et al., 2008), GI surgery (Fleckenstein et al., 2010), arthroscopic microsurgery (Toivonen et al., 2007), cholecystectomy (Puura et al., 2006), thyroid surgery (Smirnov et al., 2008) and fractional curettage in women (Phittayawechwiwat et al., 2007; Manyou and Phupong, 2008). In most instances, the administration occurred post-operatively. The analgesic effect was only moderate. On the basis of this and a former study (Renner et al., 2010), we conclude that post-operative administration of etoricoxib may not provide the full therapeutic benefit since early absorption and a reliable onset of action may be lacking due to delayed absorption (Dallob et al., 2003; Renner et al., 2010). Indeed, Fletcher et al. compared preoperative administration of ketorolac with ketorolac (at the same dose) given post-operatively. They found that pain relief was significantly superior with preoperative administration (Fletcher et al., 1995). In contrast, celecoxib given preoperatively did not provide superior post-operative pain control as compared with post-operative administration (Sun et al., 2008). However, celecoxib is known to be absorbed slowly and to show variable (low) bioavailability (Brune et al., 2010). Regrettably in the study of Sun et al. (2008), the pharmacokinetics of celecoxib was not monitored.

Further evidence in support of the preoperative administration of COX inhibitors, such as etoricoxib, comes from our previous work (Renner et al., 2010). To test the relevance of our results, we analysed possible differences between pre- and post-operative drug administration by performing graphical analyses of the PK/PD relationship in both studies (see Figure 3). In our previous study, etoricoxib was administered at the day after surgery. In both treatment groups (verum and placebo), PGE_2_ accumulated in tissue exudates during the 20 h before the first etoricoxib administration. When etoricoxib was detectable in tissue exudate the PGE_2_ concentration fell, while it remained high or even increased in placebo patients. As a result, we observed a direct PK/PD relationship between drug accumulation and inhibitory effects (Figure 3A). In our present study, etoricoxib was administered before surgery, consequently PGE_2_ concentrations were low and remained low in the verum group, but increased in the placebo group after some lag time. The delay was reflected in a counterclockwise hysteresis (Figure 3B). In accordance, we also observed a counterclockwise delay for IL6 inhibition in plasma hinting at a stimulus (surgery) related increase in cytokine release (Figure 3C). Previous and present data therefore demonstrate the inhibitory effect of etoricoxib in the ‘effect compartments’ with different lag times. The inhibition by etoricoxib on prostaglandin production becomes visible in the present study only in comparison to the delayed and lasting increase of PGE_2_ production in the control group. Preoperative administration of etoricoxib blocks the post-operative PGE_2_ production. Obviously, the early inhibition of the production of pro-algesic PGE_2_ (pre-stimulus) may result in an improved pain relief as indicated by our pain assessments.

Not all patients may benefit from an early administration of COX-2 selective inhibitors such as etoricoxib. Many patients undergoing surgery may present with contraindications of etoricoxib. Two randomized, placebo-controlled studies exist using valdecoxib and the parenteral prodrug parecoxib after coronary artery bypass surgery – now a contraindication for the use of selective COX inhibitors. In the first study by Ott et al., 462 patients were treated up to 14 days post-operatively with intravenous parecoxib or by oral valdecoxib. The authors observed a higher but not significant incidence of cardiovascular and cerebrovascular adverse events in the treatment group hinting at a thrombotic risk in these patients (Ott et al., 2003). In a further study comprising 1671 patients, both drugs (parecoxib and/or valdecoxib) were used up to 10 days after cardiac surgery and the investigators observed a significant higher incidence (0.5% vs. 2%) of cardiovascular events in one (intravenous parecoxib) of the three subgroups (Nussmeier et al., 2005). In our study with etoricoxib, patients with cardiovascular risks were excluded according to protocol. Thus, perioperative studies with a high number of patients might be necessary to demonstrate whether there are possible treatment effects on thrombotic or embolic events in patients lacking cardiovascular risks, too.

In conclusion, our data support the preoperative use of etoricoxib in patients undergoing major surgery which goes along with serious post-operative pain, provided there are no contraindications.
